# Using Rice Bran Extract (RBE) as Supplement for Mescenchymal Stem Cells (MSCs) in Serum-free Culture

**DOI:** 10.1186/1753-6561-7-S6-P58

**Published:** 2013-12-04

**Authors:** Rinaka Yamauchi, Ken Fukumoto, Satoko Moriyama, Masayuki Taniguchi, Shigeru Moriyama, Takuo Tsuno, Satoshi Terada

**Affiliations:** 1 University of Fukui, Fukui, 910-8507, Japan; 2 Niigata University, Niigata, 950-2102, Japan; 3 Tsuno Food Industrial Co., Ltd, Katsuragi-cho, Wakayama, 649-7122, Japan

## Introduction

Currently, therapies using multipotent mescenchymal stem cells (MSCs) are tested clinically for various disorders, including cardiac disease [1]. However, conventional culture media contain fetal bovine serum (FBS) and so the concern about amphixenosis remains. Therefore, developing animal derived factor-free media are desired [2].

We previously reported that rice bran extract (RBE) significantly improved the proliferation of various cell lines and the cellular functions. In this study, we tested the effect of RBE on MSCs in serum-free culture.

## Materials and methods

### Effect of RBE on osteogenic differentiation

MSCs obtained from the bone marrow of Wistar rats were cultured under conventional *α*-MEM with 15% FBS medium, supplemented with or without RBE for three days at passage 1 - 3. After treatment with RBE for three days, the media were replaced by RBE-free osteogenic medium composed of *α*-MEM containing 10% FBS, 10 mM *β*-glycerol phosphate (Merck, USA), 0.05 mM L-ascorbic acid 2 phosphate (Sigma, USA), 10 nM dexamethasone (Sigma), 1% penicillin-streptomycin solution and the cells were cultured in the medium for 24 days. To evaluate the differentiation ability, the cells were stained with Alizarin Red S and analyzed by using Image J.

### Effect of RBE on cell proliferation

After MSCs were cultured in the presence of RBE for three days, viable cell number was measured by the trypan blue dyeing assay on a hemocytometer.

### Effect of RBE on gene expression after expansion

After treatment with RBE for three days, cells were lysed to be analyzed the maintaining MSC markers with real-time PCR. Total RNA from the cells was isolated by Acid Guanidinium Phenol Chloroform method and cDNA was synthesized with supersucriptTM (Invitologen, USA). These cDNAs were analyzed by LightCycler R480 (Roche, Germany) using primers: MSC markers, CD44, CD105 and CD166, and osteogenic genes, BMP2, ALPL, OCN. The results were normalized with respect to GAPDH or HPRT. Relative mRNA quantify was calculated using the comparative ΔΔCT.

## Results and discussion

**Figure 1:**
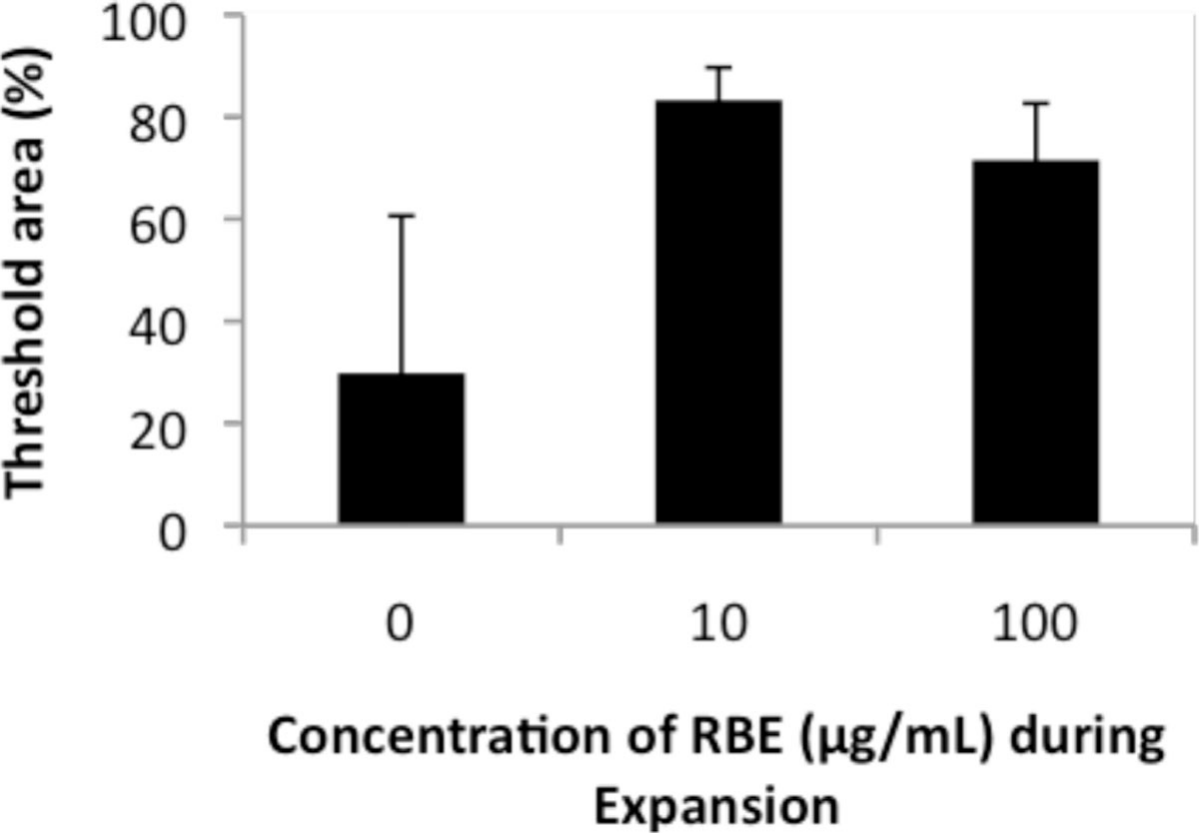
**Effect of RBE on osteogenic differentiation**.

As shown in Figure 1, threshold area (%) was significantly increased in MSCs expanded in the presence of RBE in comparison with in absence (*P < 0.03), suggesting that the cells expanded in RBE-containing medium differentiated into bone superior to the negative control cells.

The viable cell densities in the culture with and without RBE were quite similar, suggesting that increase in osteogenisis with RBE is not due to the population of the cells. Expression levels of MSC markers such as CD44, CD105 and CD166, were not up- nor down-regulated in the presence of RBE during expansion, whereas that of osteogenic gene BMP2 was remarkably reduced. These results suggest that RBE does not induce osteogenesis during expansion and imply that RBE could keep MSCs undifferentiatiated.

Treatment with RBE during expansion up-regulated the expression levels of osteogenic genes including ALPL and OCN in MSCs during osteogenic differentiation.

## Conclusion

Decreased osteogenic differentiation ability of MSCs after expansion could be maintained by addition of RBE into expansion medium. RBE is a candidate for the novel supplement for maintaining differentiation ability of MSCs in expansion culture.
